# Loureirin B, an essential component of *Sanguis Draxonis*, inhibits Kv1.3 channel and suppresses cytokine release from Jurkat T cells

**DOI:** 10.1186/2045-3701-4-78

**Published:** 2014-12-12

**Authors:** Shijin Yin, Qinglan Hu, Jialie Luo, Yuxin Li, Chunlan Lu, Xuan Chen, Hongzhen Hu

**Affiliations:** College of pharmacy, South-Central University for Nationalities, Wuhan, 430074 P R China; Center for the Study of Itch, Department of Anesthesiology, Washington University School of Medicine, St. Louis, MO 63110 USA

**Keywords:** Loureirin B, Sanguis draxonis, Kv1.3 channels, IL-2

## Abstract

*Sanguis draxonis* (SD), also known as “Dragon’s Blood”, is a traditional herb medicine that has been used to treat a variety of complications with unknown mechanisms. Recent studies show that SD displays immunosuppressive activities and improves symptoms of type I diabetes in animal models. However, the mechanisms underlying SD’s immunosuppressive actions are not completely understood. The voltage-gated Kv1.3 channel plays a critical role in the pathogenesis of autoimmune diseases by regulating the functions of both T cells and B cells. Here we investigated the effect of SD and one of its active components loureirin B (LrB) on Kv1.3. Both SD and LrB inhibited Kv1.3-mediated currents, produced a membrane depolarization, and reduced Ca^2+^ influx in Jurkat T cells. In addition, application of LrB inhibited phytohemagglutinin (PHA)-induced IL-2 release from activated Jurkat T cells. Furthermore, point mutations in the selective filter region significantly reduced the inhibitory effect of LrB on Kv1.3. The results of these experiments provide evidence that LrB is a channel blocker of Kv1.3 by interacting with amino acid residues in its selective filter region. Direct inhibition of Kv1.3 in T cells by SD and LrB might be the cellular and molecular basis of SD-mediated immunosuppression.

## Introduction

Plant-derived natural compounds not only play a critical role in ancient medicine [1] but also become unique tools to dissect disease mechanisms as well as important reservoirs of potential new drugs [2–5]. SD, also called “dragon’s blood”, is a famous herb medicine used for a variety of applications including blood stasis, oxidative stress, inflammation, tumors and immune suppression [6].

The immunomodulatory activity of SD was proposed to involve inhibition of both classical (CP) and alternative (AP) pathways of complement system as well as proliferation of activated T-cells [7], suggesting that SD might be able to inhibit autoimmune diseases. In fact, recent studies have shown that SD is effective in reducing glycemia and increasing insulin sensitivity in diabetic rats [8, 9]. Further studies demonstrated that oral application of SD reduces release of inflammatory cytokines, protects pancreas function, and markedly improves diabetic symptoms in a rat model of streptozotocin (STZ)-induced Diabetes mellitus, a well-recognized autoimmune condition [10, 11]. Although these studies have provided convincing evidence that SD is likely a potent inhibitor of autoimmune disorders, the cellular and molecular mechanisms underlying the immunomodulatory effect of SD are still poorly understood.

The pathogenesis of autoimmune diseases involves activation of effector memory T cells (T_EM_ cells) and/or class-switched memory B cells, which is evident in a number of autoimmune diseases including multiple sclerosis (MS), rheumatoid arthritis, systemic lupus erythematosus, and type-I diabetes, etc. [12–17]. Activated T_EM_ cells migrate into tissues, secret inflammatory cytokines, and contribute to deleterious inflammatory damages [18]. Memory B cells, especially those belonging to the class-switched CD27^+^IgD^-^ subset, are also involved in the pathogenesis of many autoimmune diseases [19–21].

A rise in intracellular free Ca^2+^ ([Ca^2+^]_i_) in lymphocytes is an essential signal required for release of inflammatory cytokines that precedes inflammatory damage in autoimmune diseases [22]. T cell activation relies on the operation of voltage-gated and Ca^2+^-activated K^+^ channels as well as calcium release-activated channels (CRAC) channels which are composed of *Orai* proteins [23]. Homomeric voltage-gated Kv1.3 channel is highly expressed in autoreactive T_EM_ cells from MS patients and considered as a critical therapeutic target for T cell-mediated autoimmune diseases [24]. Kv1.3 is also found in human B lymphocytes and its expression is up-regulated in class-switched memory B cells [25]. Accordingly, extensive efforts have been made to discover and develop small molecule inhibitors of Kv1.3 as novel immunosuppressants and immunomodulators [26].

SD possesses great medicinal values because of its rich flavone components that offer benefits for effective treatment of diseases [6, 27, 28]. Recent studies have shown that total flavones, especially its active component LrB, mediate the effect of SD to regulate functions of several pain-related ion channels including inhibition of voltage-gated Na^+^ currents and TRPV1-mediated cation currents in primary sensory neurons, which might contribute to the pain-soothing effect of SD [29–31]. Considering the fact that SD inhibits the proliferation of activated T-cells and Kv1.3 plays an essential role in T_EM_ cell activation, we hypothesize that SD and its active components might regulate Kv1.3 function. Here we found that SD inhibits Kv1.3-mediated currents in Jurkat T cells, and further demonstrated that its essential active component LrB directly suppresses Kv1.3 by blocking the channel pore, thereby inhibiting the release of IL-2.

## Results

### SD and LrB inhibit Kv1.3 in Jurkat T cells

**Figure 1:**
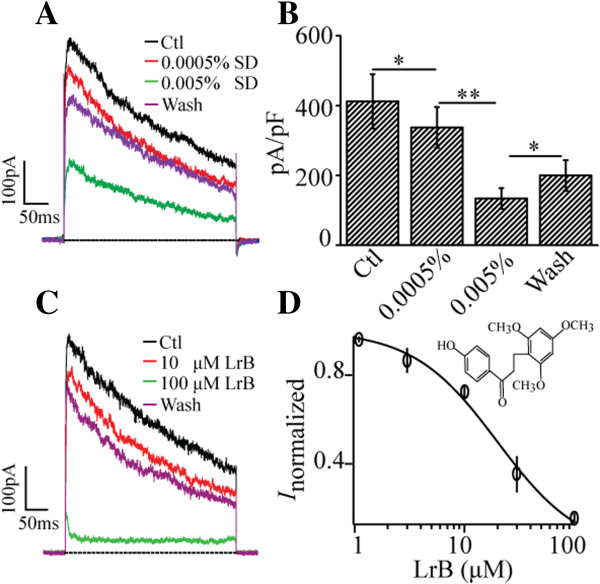
**SD and LrB inhibit Kv1.3 endogenously expressed by Jurkat T cells. (A)** Representative traces illustrate that SD inhibited the Kv1.3 current in a Jurkat T cell in a concentration-dependent manner. **(B)** Bar chart illustrates that 0.0005% and 0.005% SD significantly inhibited the Kv1.3 current density (*n* = 5, *p < 0.05, **p < 0.01). The inhibitory effect was partially recovered after washout of the drug. **(C)** Representative current traces show that LrB inhibited Kv1.3 currents in a Jurkat T cell in a concentration-dependent manner and the effect was partially reversible. **(D)** Concentration-response curve of LrB inhibition of Kv1.3 current in Jurkat T cells. Currents were normalized to the control and fitted by a Hill equation; EC_50_ value was 19.9 ± 1.8 μM and Hill slope was 1.1 ± 0.1 (*n* = 8); the structure formula of LrB was shown in the inset.

Two major types of K^+^ channels are expressed by the human Jurkat T cells: the Kv1.3 (a voltage-dependent K^+^ channel) [32], and the apamin-sensitive small conductance Ca^2+^-dependent K^+^ channel (SKCa2) which is activated by a rise in cytosolic Ca^2+^[33]. We first examined whether SD could regulate the activation of endogenous Kv1.3 expressed by human Jurkat T cells which is commonly used to study T cell signaling [32, 34]. To avoid activation of the SKCa2 channel we used a pipette solution containing almost zero cytosolic Ca^2+^. Kv1.3-mediated currents were elicited by 400 ms depolarizing pulses to +50 mV, from a holding potential of -60 mV. Bath application of SD reduced Kv1.3 current by 34.9 ± 12.9% even at a low concentration of 0.0005% (n = 5). The inhibitory effect of SD was concentration-dependent (Figure 1B) with the highest concentration of SD (0.005%) decreasing the Kv1.3 current by 73.6 ± 9.5% (n = 5) (Figure 1A). The suppressive effect of SD was partially reversible after washout (Figure 1B).

LrB is one of the flavonoids in SD and also the most potent active component of SD that regulates voltage-gated Na^+^ channels [31] and TRPV1 [29]. We therefore tested whether LrB had a similar inhibitory effect as SD on Kv1.3 in Jurkat T cells. Indeed, LrB alone reduced the Kv1.3 current in a concentration-dependent manner with an IC_50_ of 19.9 ± 1.8 μM (n = 8) (Figure 1D). Like SD, the suppressive effect of LrB was partly reversible after washout (Figure 1C). Therefore, it is very likely that LrB contributes to the inhibitory action of SD on the Kv1.3.

### LrB causes a membrane potential depolarization in Jurkat T cells

**Figure 2:**
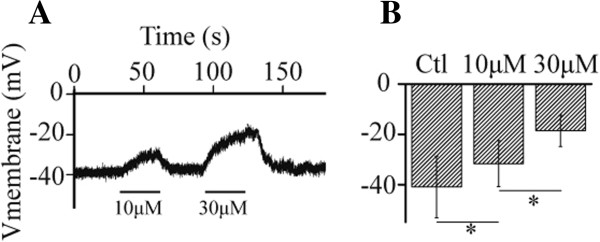
**LrB evokes a membrane depolarization in Jurkat T cells. (A)** The representative voltage trace shows that 10 and 30 μM LrB caused a depolarization of the membrane potential in a Jurkat T cell in a reversible manner. **(B)** Statistical data averaged from 10 cells (*P < 0.05).

As one of the main resting K^+^ channels, Kv1.3 is responsible for generating the resting membrane potentials in the Jurkat T cells [35]. Therefore, suppression of Kv1.3 by LrB should lead to a membrane depolarization of the Jurkat T cells. To test this hypothesis we measured the effect of LrB on the membrane potential of Jurkat T cells by means of current-clamp recording. As expected, 10 μM LrB depolarized the membrane potential of Jurkat T cells from -40.9 ± 12.3 mV to -31.7 ± 9.2 mV (n = 10). At 30 μM, LrB further depolarized the membrane potential of Jurkat T cells to -18.6 ± 6.2 mV (n = 10). The effect of LrB on the membrane potential of Jurkat T cells was partially reversible after washout (Figure 2A and B).

### LrB suppresses Ca^2+^ signaling in Jurkat T cells

As Kv1.3 is involved in generating the resting membrane potential which drives Ca^2+^ influx and contributes to Ca^2+^ homeostasis in T cells, and a previous study showed that inhibition of Kv1.3 reduces Ca^2+^ influx in Jurkat T cells [35], suggesting that LrB should also reduce Ca^2+^ influx in Jurkat T cells. To test this hypothesis we performed live cell Ca^2+^ imaging using the Ca^2+^ indicator dye Fura-2 to determine the effect of LrB on changes in [Ca^2+^]_i_ in the presence of intracellular Ca^2+^ store depletion by cyclopiazonic acid (CPA), an inhibitor of the sarcoplasmic reticulum Ca^2+^-ATPase [36, 37].

**Figure 3:**
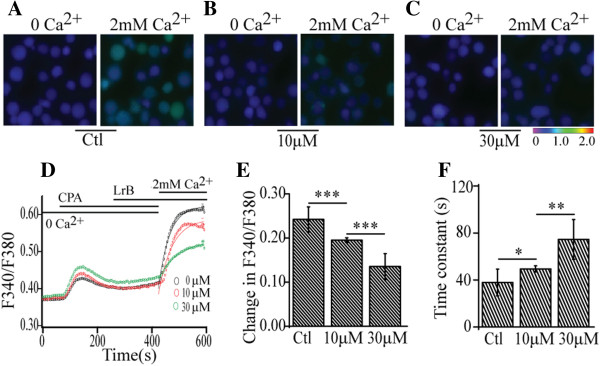
**Inhibition of Ca** ^**2+**^ **influx by LrB in Jurkat T cells. (A)** Representative images were taken in the presence of Ca^2+^-free extracellular solution (left) and 6 minutes after 2 mM Ca^2+^ was added (right) with the intracellular Ca^2+^ store depleted by 10 μM CPA. **(B)** and **(C)** Representative images illustrate that 10 and 30 μM LrB markedly decreased the intracellular Ca^2+^ response induced by 2 mM extracellular Ca^2+^ in CPA-treated Jurkat T cells. **(D)** Representative traces show the effect of LrB on store depletion-induced Ca^2+^ influx. Each trace represents averaged F340/F380 ratio from about 400 Jurkat T cells. The ratio traces in the presence of 0, 10 and 30 μM LrB are color-coded with black, red and green, respectively. The first [Ca^2+^]_i_ peak presents a rapid Ca^2+^ rise evoked by 10 μM CPA in 0 Ca^2+^ in extracellular solution, and the second [Ca^2+^]_i_ peak illustrates the sustained store depletion-induced Ca^2+^ influx with an addition of 2 mM Ca^2+^ in the absence and presence of different concentrations of LrB. The second peak is fitted with a single exponential function to calculate the rise time constant of store depletion-induced Ca^2+^ influx. **(E)** Statistical data summarizes the net changes in F340/F380 ratios induced by 2 mM extracellular Ca^2+^, ***P < 0.001. **(F)** Statistical data illustrates the rise time constant of store depletion-induced Ca^2+^ influx in the presence of 0 (Ctl), 10 and 30 μM LrB, *P < 0.05, **P < 0.01.

In the absence of extracellular Ca^2+^, 10 μM CPA induced a relative small, transient rise of [Ca^2+^]_i_ in Jurkat T cells. When 2 mM Ca^2+^ was added to the Ca^2+^-free HBSS, we observed a large Ca^2+^ influx into the cells (Figures 3A and D). Pre-treatment of Jurkat T cells with LrB for 3 min produced a concentration-dependent inhibition of the Ca^2+^ influx (Figure 3B, C, D). At 10 μM, LrB decreased the change in F340/F380 ratio from 0.24 ± 0.03 to 0.19 ± 0.01 (n = 296) (Figure 3E), and increased the rise time constant from 37.9 ± 11.4 s to 49. 4 ± 2.6 s (n = 296) (Figure 3F). At 30 μM, LrB further decreased the change in F340/F380 ratio to 0.14 ± 0.03 (Figure 3E), and increased the rise time constant to 74.5 ± 16.9 s (n = 296) (Figure 3F). These results suggest that LrB is a potent inhibitor of intracellular Ca^2+^ signaling of T cells through inhibition of Kv1.3.

### LrB inhibits IL-2 secretion from activated Jurkat T cells

**Figure 4:**
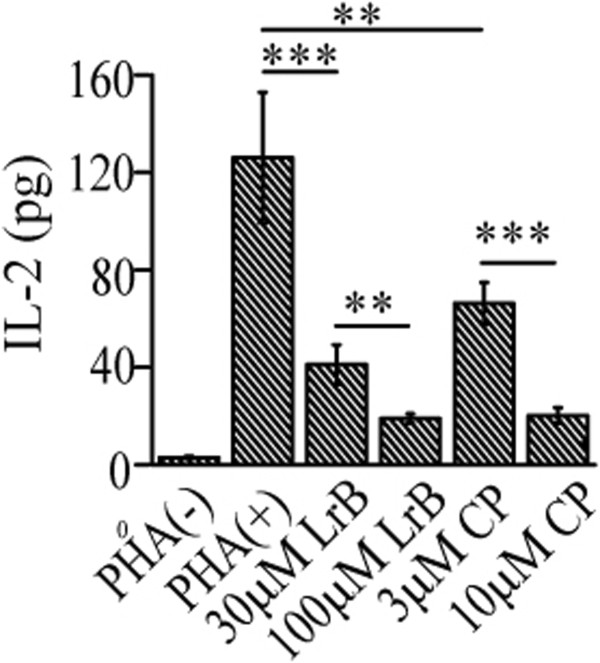
**LrB inhibits IL-2 secretion from activated Jurkat T cells.** The concentrations of IL-2 in cell growth media were measured by ELISA. Jurkat T cells were activated by PHA (10 μg/ml) for 24 hours. LrB (30 and 100 μM), CP-339818 (CP, 3 and 10 μM) were added simultaneously with PHA. **P < 0.01, ***P < 0.0001, n = 6.

The cytokine IL-2 is predominantly secreted from activated T cells and is critical in regulating the balance between immune tolerance and autoimmunity [38, 39]. The secretion of IL-2 is driven by a rise of [Ca^2+^]_i_[40]. Since LrB potently suppresses Kv1.3 function and decreases Ca^2+^ influx in Jurkat T cells we next asked whether LrB could inhibit IL-2 secretion, which should result in functional immunosuppression. As a classical polyclonal stimulator acting through T-cell receptor complex (TCR)-CD3 complex, PHA has been used to induce T cell activation and stimulate IL-2 secretion, which is dependent on an increase of [Ca^2+^]_i_[41, 42]. We thus used the PHA-stimulated IL-2 secretion in the growth media as readout. As predicted, IL-2 concentration measured by ELISA was increased markedly upon stimulation with 10 μg/ml PHA but not vehicle control (Figure 4). Pre-treatment of the cells with different concentrations of LrB (10 and 30 μM) for 24 hours significantly reduced PHA-induced IL-2 secretion in a concentration-dependent manner. A similar concentration-dependent inhibitory effect was also observed when the cells were pre-treated with CP339818, another potent Kv1.3 blocker (Figure 4) [43].

### Structural basis of LrB-mediated inhibition of Kv1.3 channel function

**Figure 5:**
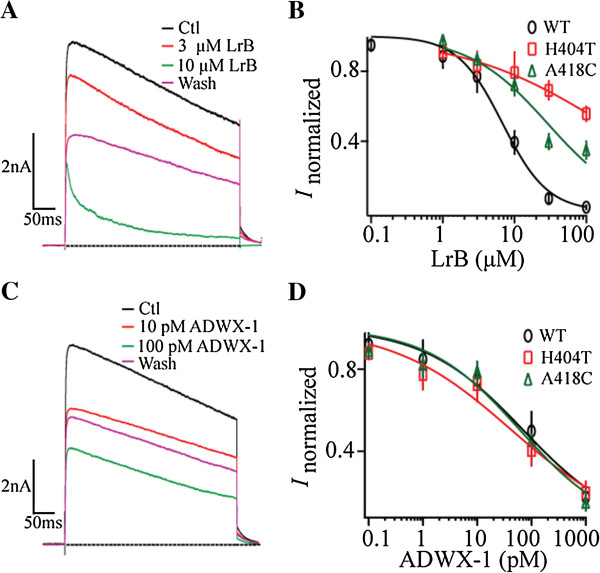
**Point mutations in the selective filter region reduced the inhibitory effect of LrB on mKv1.3. (A)** Representative mKv1.3 current traces at +50 mV in the absence (Ctl) or presence of 3 or 10 μM LrB. **(B)** Concentration-response curves of LrB inhibition on currents mediated by the wild-type or mKv1.3 mutants taken at +50 mV. Each data point represents mean ± SE of at least five experiments. **(C)** Representative mKv1.3 current traces at +50 mV in the absence (Ctl) or presence of 10 or 100 pM ADWX-1. **(D)** Concentration-response curves of ADWX-1 inhibition on currents mediated by the wild-type or mKv1.3 mutants taken at + 50 mV. Each data point represents mean ± SE of at least five experiments.

We next asked if LrB also inhibits Kv1.3 channels heterologously expressed in HEK293T cells. As expected, LrB not only reduced the peak amplitude of wild-type mKv1.3-mediated currents in a concentration-dependent manner, which recapitulates the phenomenon in the Jurkat T cells, but also changed the time course for current inactivation at high concentrations (Figure 5A). The steady-state current measured at the end of the depolarizing pulse was also markedly decreased by LrB with an IC_50_ of 7.2 ± 0.6 μM (n = 8). Interestingly, the inactivation of mKv1.3 was well fitted to a single exponential function with a time constant of 113.7 ± 3.8 ms in the absence of LrB (n = 8) but displayed a double exponential function with both fast (5.2 ± 0.4 ms) and slow (69.7 ± 5.3 ms, n = 8) components in the presence of 10 μM LrB (Figure 5A).

The effect of LrB to accelerate the decay time of Kv1.3 channels is reminiscent of the effect of verapamil on Kv1.3 [44], suggesting that LrB might share the same binding sites with verapamil in the recombinant Kv1.3 channels. To test this hypothesis, we constructed two mKv1.3 mutants in which the verapamil binding sites are disrupted [44]. In one of the mutants we replaced His404 with a Thr and in the other mKv1.3 mutant Ala418 was replaced with a Cys.

In HEK293T cells expressing mKv1.3-H404T, LrB suppressed the steady-state current with an IC_50_ of 116.5 ± 19.8 μM (n = 8), 16.2 times higher than that of the wild-type mKv1.3. For mutant Kv1.3-A418C, the IC50 of LrB inhibition of the steady-state current was 29.1 ± 7.1 μM (n = 8), about 4 times higher than that for the wild-type mKv1.3 (Figure 5B). Interestingly, ADWX-1, a potent and selective peptide inhibitor of Kv1.3 acting through binding to the extracellular pore turret of the Kv1.3 [45], had a comparable inhibitory effect on wild-type mKv1.3, mKv1.3-H404T, and mKv1.3-A418C mutants (Figure 5C and D). These data suggest that LrB, like verapamil, is a deep pore blocker of the mKv1.3, presumably interacting directly through His404 and A418 residues.

## Discussion

SD is one of the rare traditional herb remedies that have been widely used in clinical practice because of its effectiveness in treating a variety of diseases [6]. SD possesses complex components and displays a variety of pharmacological activities. Recent exciting studies have significantly advanced our knowledge about the pharmacological activities of SD [46, 47]. Further studies using electrophysiological recordings have revealed that LrB, cochinchinenin A and cochinchinenin B are the three main active ingredients of SD and potentially mediate SD’s analgesic effect by inhibiting voltage-gated Na^+^ channels and the pain-initiating TRPV1 channels in the primary sensory neurons [47–50]. In addition to its analgesic effect, SD’s immunomodulatory activity has also gained widespread attention in recent years [51, 52] and is considered as the main reason for its effectiveness in the treatment of diabetes [11]. However, unlike its analgesic effect, the molecular targets of SD’s immunomodulatory effect have not been identified and the active ingredients mediating the immunomodulatory effects have not been determined. Here we show that both SD and its active component LrB have an inhibitory effect on Kv1.3 which regulates the function of lymphocytes. LrB also inhibits the release of inflammatory cytockine, IL-2, from the activated Kv1.3-expressing Jurkat T cells. Furthermore, we show that two amino acid residues in the inner pore region are required for the inhibitory action of LrB on Kv1.3. Our results suggest that LrB and its analogs might exert their immunomodulatory effects by directly blocking the Kv1.3 channels expressed by both T and B lymphocytes.

Autoimmune diseases occur when the Kv1.3 expression level increases sharply in the proliferating T_EM_ cell membrane [53]. Indeed, Kv1.3 blockers can inhibit the activation and proliferation of the T_EM_ cells [54]. There is also a growing body of evidence suggesting that Kv1.3 channel blockers have beneficial therapeutic effect on rheumatoid arthritis [55], autoimmune encephalitis [54] and other autoimmune diseases. Consistent with being a Kv1.3 inhibitor, SD effectively reduces islet β cell damage in a rodent model of STZ-induced type I diabetes and increases levels of plasma insulin, thereby lowering blood glucose [10], suggesting that SD has a therapeutic effect on autoimmune diseases. *In vitro* studies show that SD also inhibits the activation and proliferation of T lymphocytes [51], excessive activation of which is associated with autoimmune diseases. Since T_EM_ cell proliferation is directly associated with the pathogenesis of autoimmune diseases, these findings strongly suggest that therapeutic benefit of SD on autoimmune diseases likely results from inhibition of the activation and proliferation of T lymphocytes [53]. In this study, we demonstrate that SD has a blocking effect on Kv1.3 channels endogenously expressed by the human Jurkat T lymphocytes. LrB, the main active ingredient of SD, can also block the Kv1.3 channel, which is reminiscent of its inhibitory action on the voltage-gated Na^+^ channels expressed by the primary sensory neurons [31]. These findings not only help to explain the immunomodulatory activity of SD, but might also provide a molecular basis for its contribution to increased insulin sensitivity and improvement of type I diabetes [56].

Similar to other Kv1.3 blockers, application of LrB acutely depolarizes the cell membrane potential of Jurkat T cells (Figure 2) [35], which will weaken driving force for the extracellular Ca^2+^ influx and reduce the [Ca^2+^]_i_ in Jurkat T cells (Figure 5) [57]. It is well-known that an increase of [Ca^2+^]_i_ level is directly associated with up-regulated transcription and release of inflammatory mediators including IL-2, TNFα, etc. [58]. Therefore, Kv1.3 channel blockers can inhibit the release of inflammatory mediators through suppressing the [Ca^2+^]_i_ increase in Jurkat T cells [57, 59]. Our studies found that LrB exerts many similar effects as other Kv1.3 channel blockers [35, 57], for instance, inhibition of PHA-induced IL-2 release from the Jurkat T cells. These findings further support that LrB might be an active immunoregulatory ingredient of SD.

With the establishment of Kv1.3 channel as an excellent drug target for autoimmune diseases [53], extensive efforts have been made to develop selective and efficient Kv1.3 channel blockers and provide lead drugs for the treatment of autoimmune diseases [26, 60, 61]. Currently, Kv1.3 channel blockers have two main sources: 1) polypeptide toxins (20–50 amino acid residues) mainly from animal poisonous glands such as in scorpions [60], anemones [62]. Most of these peptide toxins block the channel mouth (outer vestibular) to inhibit Kv1.3 [45]; 2) small organic molecules mainly from medicinal plants. They usually bind to the inner pore region of Kv1.3 and serve as open channel blockers to accelerate the current decay [44]. Our studies show that LrB produces a concentration-dependent inhibition of mKv1.3 heterologously expressed by HEK293T cells and displays characteristics of small molecule blockers by accelerating the current decay of mKv1.3 channels. The blocking effect of LrB shares certain similarities as that of another Kv1.3 blocker verapamil and might share some common verapamil recognition sites located in the selectivity filter region of Kv1.3 since mutants carrying mutations disrupting key verapamil interaction site also significantly attenuate LrB inhibition of Kv1.3 [63].

In summary, the current study demonstrates that the traditional herb medicine SD and its main active ingredient LrB are blockers of Kv1.3 channels. We also show that LrB causes a membrane depolarization and inhibits IL-2 release from the Jurkat T cells. Furthermore, we have identified two amino acid residues on the inner pore of mKv1.3 that are critical to LrB inhibition. These findings provide molecular and cellular basis of the immunomodulatory activities mediated by SD and its active component LrB.

## Materials and methods

### Cell culture, vector constructs, and transfection

Jurkat E6-1 T cells (ATCC TIB152) and HEK293T cells (ATCC ACS4500) were maintained in RPMI medium 1640 (Invitrogen, Carlsbad, CA, USA) and Dulbecco's modified Eagle's medium (DMEM) (Life Technologies, GrandIsland, NY, USA), supplemented with 10% fetal bovine serum (Life Technologies), 100 units/ml penicillin, 100 μg/ml streptomycin, respectively. Cells were cultured in a humidified incubator at 37°C with 5% CO_2_. cDNAs encoding mKv1.3 in pSP64 (a generous gift from Prof. Stephan Grissmer, University of Ulm, Ulm, Germany) were subcloned into the XhoI/BamH I sites of pIRES2-EGFP (Clontech, Inc., Mountain View, CA, USA). All mKv1.3 mutants were made using QuikChange II XL mutagenesis kit (Agilent Technologies, Inc., Santa Clara,CA, USA) according to manufacturer's directions and confirmed by DNA sequencing. HEK293T cells were transiently transfected with wild-type and individual mKv1.3 mutants using Lipofectamine 2000 (Invitrogen) and maintained in DMEM at 37°C for 24 hrs before experiments.

### Solutions and chemicals

The External solution used to record Kv1.3 currents contained (in mM): 5 KCl, 140 NaCl, 10 Hepes, 2 CaCl_2_, 1 MgCl_2_, and 10 D-glucose (pH 7.4 with NaOH). The internal solution contained (in mM): 140 KCl, 1 MgCl_2_, 1 EGTA, 3 Na_2_ATP, and 10 Hepes (pH 7.2 with KOH). ADWX-1 was from Wuhan More Biotechnology Co, Ltd (Wuhan, China) and SD and LrB were from Shanghai Pure One Biotechnology (Shanghai, China). All other chemicals were from Sigma (St. Louis, MO, USA).

### Electrophysiology

Whole-cell patch-clamp recordings were performed using an EPC 10 amplifier (HEKA Elektronik, Lambrecht/Pfalz, Germany) at room temperature (22-24°C). Pipettes pulled from borosilicate glass (BF 150-86-10; Sutter Instrument Company, Novato, CA, USA) had resistances of 2–4 MΩ when filled with the internal solution. Kv1.3 currents were elicited by a +50 mV, 400 ms depolarizing pulse from a holding potential of -60 mV every 30 s. The membrane potential was measured in zero current (I = 0) model using whole-cell current-clamp technique.

### Live cell Ca^2+^ imaging

Jurkat T cells were loaded with 4 μM Fura-2 AM (Life Technologies) for 60 min at 37°C. Cells were then washed 3 times and incubated in Hank's Balanced Salt Solution (HBSS) for 30 min at room temperature before use. Fluorescence at 340 nm and 380 nm excitation wavelengths was recorded on an inverted Nikon Ti-E microscope equipped with 340, 360 and 380 nm excitation filter wheels using NIS-Elements imaging software (Nikon Instruments Inc., Melville, NY, USA). Fura-2 ratios (F340/F380) reflect changes in [Ca^2+^]_i_ upon stimulation. Data were obtained from 100–250 cells in time-lapse images from each coverslip.

### IL-2 secretion measurements

IL-2 secretion from Jurkat T cells was measured using an ELISA kit (eBioscience, San Diego, CA, USA) following manufacturer's instructions. Cells were centrifuged at 1500 rpm for 10 min, and the supernatants were collected to measure IL-2 concentrations. Reactions were performed in 96-well plates coated with the capture antibody and stopped with phosphoric acid (1 M). Absorbance was measured at 450 nm. Each experiment was repeated at least three times in duplicate.

### Statistical analysis

All data are presented as mean ± SEM for n independent observations. Statistical analysis of differences between groups was carried out using paired *t*-test or ANOVA. P < 0.05 was considered significantly different.
